# Microbial succession and microclimatic fluctuation in purple chromatic anomaly on Terracotta Warriors Pit 1

**DOI:** 10.1016/j.isci.2026.116717

**Published:** 2026-07-10

**Authors:** Qiang Luo, Zinan Yang, Ping Zhou, Fasi Wu, Xiaofen Mao, Dongpeng He, Na Xi, Jie Li, Yin Xia, Hua Li, Maosheng Shen, Xinghua Ding

**Affiliations:** 1 College of History and Culture, Hunan Normal University, 36 Lushan Road, Changsha, Hunan 410000, People's Republic of China; 2 Emperor Qinshihuang’s Mausoleum Site Museum, Qinling North Road, Xi’an, Shaanxi 710600, People's Republic of China; 3 Key Scientific Research Base of Ancient Polychrome Pottery Conservation of State Administration of Culture Heritage, Qinling North Road, Xi’an, Shaanxi 710600, People's Republic of China; 4 National Research Center for Conservation of Ancient Wall Paintings and Earthen Sites, Department of Conservation Research, Dunhuang Academy, Dunhuang, Gansu 736200, People's Republic of China

**Keywords:** Terracotta Warriors Pit No.1, Purple contamination, Continuous ambient surveillance, chromogenic activity, troglophilic cyanobacteria

## Abstract

A purple chromatic anomaly emerged on the earthen surfaces of UNESCO Terracotta Warriors Pit No. 1. Integrating microscopy, 16S/18S rRNA sequencing, and microclimatic monitoring, we identified specialized troglophilic cyanobacteria (*Iphinoe* sp. LOS-B1 and the *Oscillatoriales* EcFYyy-200 clade) as the dominant colonizers (>60% of surficial mats, up to 70% at epicenters). These taxa, previously known only from calcareous subterranean habitats, displayed violet-granulated cytoplasm, cylindrical-torulose cells (5–7 × 6–10 μm), and sheath-mediated hormocyst release—first documented on earthen heritage. Microclimatic data revealed a transient winter window (January–March 2023) with elevated illuminance (600–7,200 lux), frequent low humidity (relative humidity [RH] < 45% on 42.3% of days), and stable cool temperatures (−4°C to 10°C). This regime created a photic niche, enabling the rapid expansion of pre-adapted cave cyanobacteria. These findings demonstrate that semi-enclosed heritage sites can experience localized microbial outbreaks under specific microclimatic regimes, highlighting the need for integrated environmental and microbiological monitoring in conservation.

## Highlights

• Purple chromatic anomaly on earthen heritage at UNESCO Terracotta Army Pit 1
• Cyanobacteria *Iphinoe* and *Oscillatoriales* dominate surficial mats (>60%, up to 70%)
• Transient microclimate window: 600–7,200 lux, RH < 45% on 42.3% of days, −4°C to 10°C
• Transient environmental window facilitated invasion by pre-adapted cyanobacteria

## Graphical abstract

**Figure:**
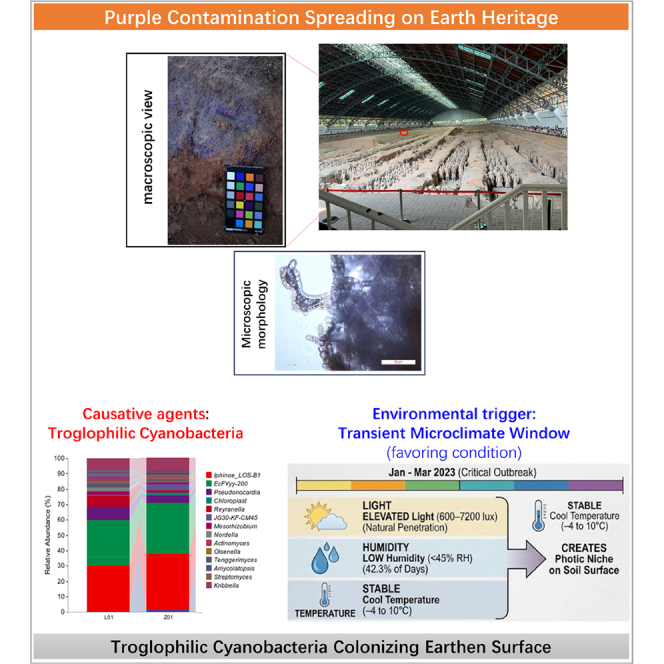

## Teaser

Social sciences; Archeology

## Introduction

Pit No. 1 of the Terracotta Army, the largest and most representative burial pit within the mausoleum complex of Emperor Qin Shi Huang, is one of the most important archaeological discoveries of the 20th century. The conservation of the Terracotta Army pits of Emperor Qin Shi Huang exemplifies *in situ* preservation, which is the primary approach adopted by most site museums in China for protecting monumental earthen remains.^1^ Pit No. 1 is covered by a steel arch canopy specifically designed for the conservation of large-scale sites.^2^ Because of the display needs of the site museum, the conservation canopy incorporates lighting and ventilation windows, and the perimeter of Pit No. 1 surrounds the viewing platform that can offer visitors convenient access for observation^3^ (Figure S1 in supplemental information). In an ideal scenario, the site museums constructed directly above archaeological remains shield the site from external influences, maintaining a stable preservation environment. However, the impact of human activities such as visits on this monumental underground earthen chamber can lead to destabilization.

**Figure 1:**
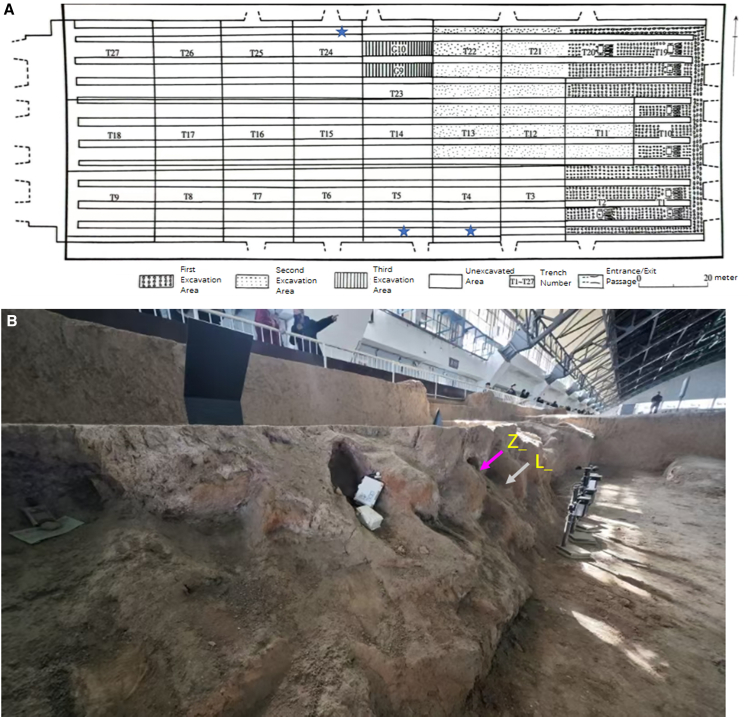
Layout of the Terracotta Warriors Pit No. 1 site and the microclimate monitoring station deployment (A) Layout of the Terracotta Warriors Pit No. 1 site. Areas affected by the anomalous purple contamination are marked with asterisks, including the pit walls of grids T4, T5, and T24. (B) A real-time microclimate monitoring station deployed within the T5 excavation grid for environmental data collection, adjacent to areas exhibiting outbreaks of purple-hue chromatic anomalies. Sunlight can directly enter the affected concave areas in grid T5 of Pit No. 1 through the ventilation windows in winter months. The arrows indicate sampling locations: the purple arrow points to the epicenter area of the purple anomaly (Z_), and the gray arrow points to the advancing margin area of the purple anomaly (L_).

Earthen heritage sites are highly susceptible to microbial colonization.^4^ The proliferation of microorganisms on earthen heritage structures leads to microbially mediated chromatic alterations through pigment production, which are manifested predominantly as dark green to brownish-black hues that obscure the original soil substrate and severely compromise the aesthetic integrity of the site.^5^ However, routine monitoring records from September 2022 documented the emergence of an atypical biological staining phenomenon characterized by an unusual purple chromatic alteration on specific surfaces within grids T4 and T5 of Pit No. 1 in the Terracotta Army. From January 2023 to April 2023, the purple discoloration spread rapidly within the T4 and T5 grids and even extended into the adjacent T24 grid area (Figure 1A). After April 2023, the purple chromatic anomaly showed no further tendency to expand and remained in a relatively stable state. *In situ* field observations recorded that the abnormal purple contamination exhibited the morphological characteristics of silvery-white to purple microbial mats. The coverage of this microbial mat changed the natural color of soil substrates in this area, causing microbial-induced chromatic anomaly (purple hue) outbreaks in a specific area within this monumental underground chamber. Similar purple contamination has usually been reported on calcareous substrates in various geographically isolated subterranean settings, including caves, hypogean environments, and catacombs.^6^^,^^7^^,^^8^^,^^9^ These troglophilic purple microflora/microbial colonizations shared similar phenotypic and ecophysiological traits; they formed purple-red filamentous mats that were partially endolithic and crept on calcareous substrates in stable light-illuminated areas as a silvery-white to purple coating. The subterranean environment on calcareous surfaces represents a confined and stable biotope, distinguished by consistent relative humidity (RH) and air temperature (TEMP), where light intensity serves as the crucial limiting factor for microbial colonization and succession on stone. Despite the generally low-light conditions in subterranean environments where these purple-red filamentous mats were discovered, phototrophic microhabitats can still develop that support the colonization, survival, and succession of microbiomes in areas where sufficient natural light penetrates or where artificial lighting is present.^6^^,^^10^

Although Pit No. 1 is similarly a subterranean structure, with its base extending 4.5–6.5 m below ground level,^11^ this study represents the first report of purple contamination on the soil surface of an earthen heritage site. To our knowledge, no similar anomalous purple contamination has previously been documented on earthen heritage sites. We therefore hypothesized that a specific and transient microclimatic regime was favorable to the development of purple microbial colonization during the outbreak period in the affected areas of Pit No. 1. In this preliminary study, we combined morphological, molecular, and microclimatic analyses to characterize the taxonomic composition of the microbiome associated with the purple discoloration, identify the putative causative microbial taxa, and establish potential links between the outbreak of this chromatic anomaly and specific microclimatic parameters.

## Results

### Microscopic morphology observation of the chromatic anomaly

**Figure 2:**
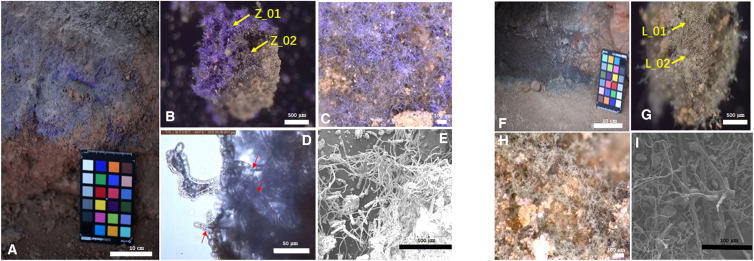
Morphological characterization of the microbiome across the epicenter area (exhibiting a vivid purple hue) and the advancing margin area of the purple anomaly in Pit No. 1 of the Terracotta Warriors (A) *In situ* macroscopic view of the purple anomaly epicenter area showing vivid purple discoloration (scale bars, 10 cm). (B) Ultra-depth-of-field micrograph of a crust sample from the purple anomaly epicenter area, showing microbial mat structures overlying the surface (Z_01) and infiltrating the underlying soil stratum (Z_02) (scale bars, 500 μm). (C) *In situ* portable micrograph of the purple anomaly epicenter area (scale bars, 100 μm). (D) High-resolution light micrograph of the surficial microbial mat from the purple anomaly epicenter area, revealing filamentous morphology. Red arrows indicate branchpoint cell structures (scale bars, 50 μm). (E) Scanning electron micrograph of the crust sample from the purple anomaly epicenter area, showing microbial mat structures overlying and infiltrating the soil stratum (scale bars, 100 μm). (F) *In situ* macroscopic view of the advancing margin area of the purple anomaly, presenting a dull gray appearance (scale bars, 10 cm). (G) Ultra-depth-of-field micrograph of a crust sample from the advancing margin area of the purple anomaly, showing microbial mat structures overlying the surface (L_01) and infiltrating the underlying soil stratum (L_02) (scale bars, 500 μm). (H) *In situ* portable micrograph of the advancing margin area of the purple anomaly (scale bars, 100 μm). (I) Scanning electron micrograph of the crust sample from the advancing margin area of the purple anomaly, showing microbial mat structures overlying and infiltrating the soil stratum (scale bars, 100 μm).

Field investigations employing a handheld digital microscope (200× magnification) revealed the morphology of the chromatic anomaly across the chromatic anomaly hotspots (epicenters exhibiting purple hue) and their advancing marginal zones to document the spatial pattern between spatial position and visual characteristics of the chromatic anomaly (Figures 2A, 2C, 2F, and 2H). We found that filamentous microbial colonization formed a cohesive mat on the soil stratum surface, exhibiting partial infiltration into the substrate (Figures 2B and 2G). The specimen from the chromatic anomaly epicenters (assigned as Z_ sample) exhibited pronounced chromatic stratification, with the surficial layer demonstrating a vivid bright purple color, while the subsurface layer maintained reduced chromogenic activity (Figure 2B). The specimen from the advancing periphery zones of this chromatic anomaly (assigned as L_ sample) exhibited a homogeneous chromatic profile in the filamentous microbial colonization of the mat matrix presenting a dull gray overall (Figure 2G). Scanning electron microscopy (SEM) analysis revealed distinct micromorphological differences between the microbial communities in the core zone and the advancing marginal zone of the chromatic anomaly. The microbial communities in the advancing marginal zone of the chromatic anomaly were primarily characterized by filamentous frameworks (15–30 μm in diameter), interwoven with finer, smooth-coated, hypha-like filaments (approximately 2–5 μm in diameter) (Figure 2I). In contrast to the homogeneous morphological profile observed in the marginal zone, the community within the chromatic anomaly epicenters exhibited characteristic filamentous architectures. These included (1) stratum-proximal, hypha-like filaments with smooth coatings, occasionally interwoven by slender dendritic filaments, displaying thigmotropic growth along soil stratum interfaces; and (2) surface-adherent filaments showing progressive hyphal inflation (with a diameter gradient of 6–10 μm) and rough textures indicative of cellular differentiation and diversification within the filamentous microbial mat matrix (Figure 2E).

Under light microscopy (LM), the surface-adherent microbial filaments within the filamentous mat at the epicenter of the chromatic anomaly appeared variously bent and entangled. Morphologically, the filaments were uniseriate and surrounded by a colorless sheath. Internally, they were composed of cylindrical to torulose cells, measuring 5–7 μm in width and 6–10 μm in length, and contained violet-colored, granulated cytoplasm. These filaments formed a loose, mat-like architecture with frequent branching, and the branchpoint cells were discernible. Furthermore, hormocyst release was observed to occur through sheath enlargement between neighboring cells; these hormocysts were occasionally formed at the ends of the main filaments (Figure 2D).

### Community diversity traits

**Figure 3:**
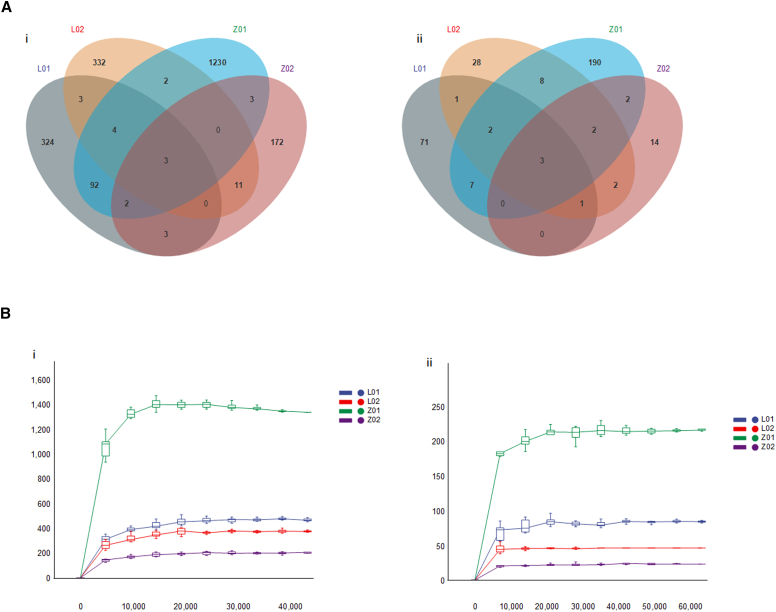
Shared and unique ASVs among samples across purple chromatic anomaly zones and rarefaction curves confirming sequencing depth sufficiency (A) The number of unique and shared amplicon sequence variants (ASVs) among samples by Venn diagram for depicting spatial heterogeneity of (i) prokaryotic consortia and (ii) eukaryotic consortia across the purple hue chromatic anomaly in Pit 1. (B) Rarefaction curves depict the number of ASVs across a range of sequencing depths for (i) prokaryotic consortia and (ii) eukaryotic consortia within each sample and enable comparison of ASV richness among samples at equivalent sequencing depths (Z01 and L01—samples of surficial mats across the chromatic anomaly epicenters and advancing marginal zones; Z02 and L02—samples of the subjacent soil strata across the chromatic anomaly epicenters and advancing marginal zones).

Sequencing of the 16S rRNA (V3–V4) and 18S rRNA (V4) gene amplicons yielded rarefaction curves approaching saturation (Figure 3B). This demonstrates that the sequencing depth was sufficient to capture the full diversity, providing a robust foundation for subsequent analyses. A summary of sequencing depth for each library is provided in Table S2. We characterized the biodiversity signatures of prokaryotic and eukaryotic consortia of _01 (the surface-adherent microbial mat) samples and _02 (the subjacent substrates of soil stratum) across the chromatic anomaly core zones and their periphery. The diversity of prokaryotic and eukaryotic consortia richness and evenness characteristics was explained by Chao1, Observed_ASVs, Shannon, and Simpson indices, which were calculated based on the amplicon sequence variant (ASV) data. The diversity results are shown in Table S1. The prokaryotic assemblages within the surface-adherent microbial mat of the purple epicenter (Z_01) exhibited hyper-diversity (Chao1 = 1,338.94), surpassing those of the other samples by 283%–651%. The Shannon-Wiener diversity index, integrating both taxonomic richness and evenness, peaked (Shannon = 4.65) in surficial mat consortia at the purple epicenters (Z_01). Secondary values (Shannon = 3.93) were recorded in advancing periphery surficial mat specimen (L_01), while attenuation occurred in the subjacent substrates of earthen stratum (Z_02: 1.01; L_02: 2.04), demonstrating vertical-dependent diversity gradients across the chromatic anomaly zones. For eukaryotic richness and evenness, the eukaryotic consortia in the surficial mat at the purple epicenters (Z_01) exhibited elevated diversity, with Chao1 richness estimates reaching 217.18 and Shannon-Wiener entropy indices of 2.42, surpassing the values of the other sites in this study. In addition, the Chao1 index value of the specimen from the surficial mat in the advancing marginal zone (L_01) was 84.87, which is higher than the values of 47 for L_02 and 24.1 for Z_02; however, the Shannon index value of the specimen from L_01 was 1.05, which is lower than the values of 1.99 for L_02 and 1.2 for Z_02 (Table S1 in supplemental information).

The Venn diagram indicates the numbers of common prokaryotic and eukaryotic ASVs among the _01 (surface‑adherent microbial mat) and _02 (subjacent soil stratum) samples across the chromatic anomaly (purple hue) in Pit 1. This diagram delineates the spatial heterogeneity across chromatic anomaly (purple hue). There were 101 common prokaryotic ASVs between the surficial mat samples (Z_01 and L_01), and the numbers of unique prokaryotic ASVs were 1,230 in the surficial mat sample at the epicenter of purple pigmentation (Z_01) and 324 in the surficial mat sample at the advancing margin of purple pigmentation (L_01). There were 14 common prokaryotic ASVs between the subjacent soil stratum samples (Z02 and L02), and the numbers of unique prokaryotic ASVs were 172 in the soil stratum sample at the epicenter of purple pigmentation (Z_02) and 332 in the soil stratum sample at the advancing margin of purple pigmentation (L_02) (Figure 3A). The Venn diagram indicated that there were 12 common eukaryotic ASVs between the surficial mat samples (Z_01 and L_01), and the numbers of unique eukaryotic ASVs were 190 in the surficial mat sample at the epicenter of purple pigmentation (Z_01) and 71 in the surficial mat sample at the advancing margin of purple pigmentation (L_01). There were 8 common eukaryotic ASVs between the subjacent soil stratum samples (Z_02 and L_02), and the numbers of unique eukaryotic ASVs were 14 in the soil stratum sample at the epicenter of purple pigmentation (Z_02) and 28 in the soil stratum sample at the advancing margin of purple pigmentation (L_02) (Figure 3A).

### Community structures of prokaryotic and eukaryotic consortia

**Figure 4:**
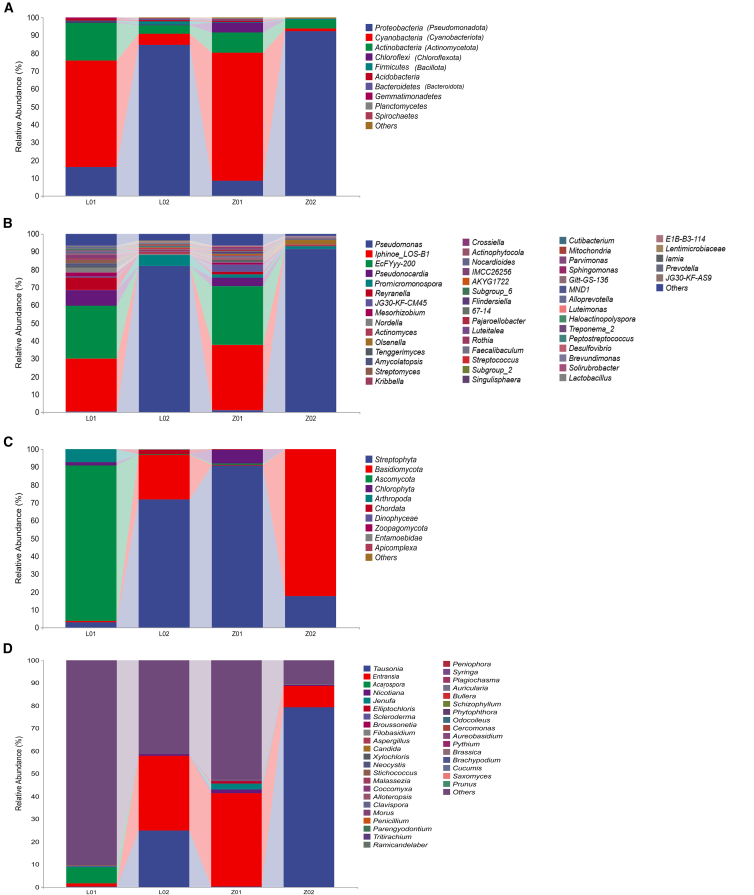
Abundance distribution of prokaryotic and eukaryotic consortia at phylum and genus levels across purple chromatic anomaly zones (A) Abundance distribution of prokaryotic consortia at the phylum level across both the chromatic anomaly (purple hue) core zone and marginal zone; the updated nomenclature for bacterial phyla as presented in parentheses has been proposed by the International Committee on Taxonomy of Prokaryotes (ICNP) in 2021. (B) Abundance distribution of prokaryotic consortia at the genus level across both the chromatic anomaly (purple hue) core zone and marginal zone. (C) Abundance distribution of eukaryotic consortia at the phylum level across both the chromatic anomaly (purple hue) core zone and marginal zone. (D) Abundance distribution of eukaryotic consortia at the genus level across both the chromatic anomaly (purple hue) core zone and marginal zone (Z01 and L01—samples of surficial mats across the chromatic anomaly epicenters and advancing marginal zones; Z02 and L02—samples of the subjacent soil strata across the chromatic anomaly epicenters and advancing marginal zones).

Through the taxonomic analyses, the top five abundant prokaryotic phyla were Proteobacteria (51%), Cyanobacteria (34%), Actinobacteria (11%), Chloroflexi (2%), and Firmicutes (1%). At the phylum level, Cyanobacteria was the most abundant prokaryotic phylum within the surficial mats across the chromatic anomaly epicenters and advancing marginal zones (Z_01 and L_01), while exhibiting a marked decline in the subjacent soil strata (Z_02 and L_02) (Figure 4A). In contrast, Proteobacteria was the most abundant prokaryotic phylum within the subjacent soil strata (Z_02 and L_02), while exhibiting a marked decline in the surficial mats across the chromatic anomaly epicenters and their advancing marginal zones (Z_01 and L_01). In addition, the relative abundance of reads affiliated with Chloroflexi in the prokaryotic reads within Z_01 accounted for 5.5%, which was higher than that in L_01 (0.9%), L_02 (0.2%), and Z_02 (0%). Taxonomic profiling revealed that the Cyanobacteria in this study were primarily represented by the taxa *Iphinoe* sp. LOS-B1 and EcFYyy-200 as keystone Cyanobacteria taxa exhibiting high abundance in the surficial mat consortia (Z_01 and L_01). These two taxa under Cyanobacteria account for over 60% of the prokaryotic reads of both the surficial mat samples (Z_01 and L_01) and even up to 70% in Z_01 (Figure 4B). The Proteobacteria was primarily composed of the genera *Pseudomonas*, *Reyranella*, *Mesorhizobium*, and *Nordella*. *Pseudomonas* demonstrated the highest abundance within the prokaryotic consortia in both Z_02 (91.7%) and L_02 (82.2%), whereas their ecological representation exhibited a marked reduction to <1% in both surficial mats (Z_01 and L_01). On the other hand, the reads affiliated with *Reyranella*, *Mesorhizobium*, and *Nordella* exhibited higher abundance in the superficial mats (Z_01 and L_01) than in the subjacent soil strata (Z_02 and L_02) samples (Figure 4B).

For the eukaryotic consortia associated with the chromatic anomaly (purple hue), *Streptophyta* and *Basidiomycota* together accounted for the highest proportion of eukaryotic lineages within the subjacent soil strata samples (Z_02 and L_02), collectively representing >95% of the total relative abundance of the eukaryotic community. *Streptophyta* and *Chlorophyta* collectively constituted >98% of the eukaryotic consortia within the surficial mat of epicenter Z_01. Within the advancing marginal zone of the surficial mat (L_01), *Ascomycota* alone constituted 87.09% of the eukaryotic consortia in this specimen (Figure 4C). At the genus level, the results showed that a large number of sequences belonged to unclassified or no rank genera based on the current database of 18S rRNA gene sequences. In this study, the phylum *Streptophyta* was primarily composed of reads affiliated with *Entransia*. The relative abundance of *Entransia* displayed the trend Z_01 > L_02 > Z_02 > L_01. The phylum *Basidiomycota* was primarily composed of reads affiliated with *Tausonia*. The relative abundance of *Tausonia* accounted for 79.34% in Z_02, higher than that in L_02 (24.91%), L_01 (0.17%), and Z_01 (0.11%). *Chlorophyta* was primarily composed of *Xylochloris*, *Coccomyxa*, *Stichococcus*, *Elliptochloris*, and *Jenufa*. These genera under *Chlorophyta* were detected only in the surficial mat specimens from the epicenters of chromatic anomaly (Z_01) (Figure 4D).

**Figure 5:**
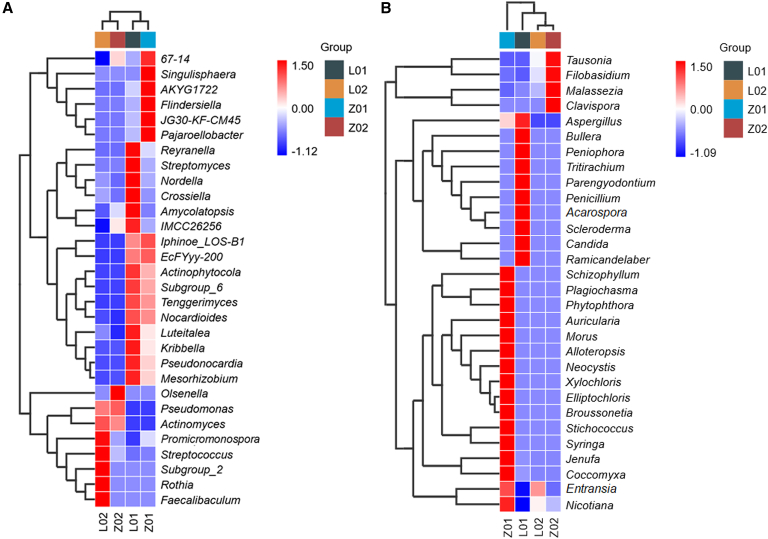
Clustering heatmaps of prokaryotic and eukaryotic consortia at genus level across purple chromatic anomaly zones (A) Prokaryotic consortia. (B) Eukaryotic consortia. Clustering analysis (heatmap) using the average linkage method was performed on the rarefied feature table using the Bray-Curtis distance metric. Rows represent how samples relate by community composition, as arranged by the horizontal dendrogram. The color of each box represents the abundance (normalized by taking the log10 value of feature table relative abundances) of each taxon present within each sample—dark colors indicate lower relative abundance, and lighter colors indicate higher relative abundance of each taxa within each sample. Clustering was performed on the rarefied feature table summarized to taxonomy annotation of the genus level, but only the top 30 genus of the total microbiome are displayed (Z01 and L01—samples of surficial mats across the chromatic anomaly epicenters and advancing marginal zones; Z02 and L02—samples of the subjacent soil strata across the chromatic anomaly epicenters and advancing marginal zones).

The heatmaps were constructed using hierarchical cluster analysis to assess the prokaryotic and eukaryotic consortia across samples (Figures 5A and 5B). For the top 30 genera distribution patterns in prokaryotic consortia, 10 taxa groups displayed higher abundance in the surficial mats (Z_01 and L_01), while exhibiting a marked decline in the subjacent soil strata (Z_02 and L_02). The genera *Pseudomonas* and *Actinomyces* exhibited higher relative abundance in the subjacent soil strata (Z_02 and L_02), while exhibiting a marked decline in the surficial mats (Z_01 and L_01). As for the eukaryotic consortia, the genus *Acarospora* under Ascomycota displayed higher abundance in the surficial mat eukaryotic consortia (L_01), while exhibiting a decline in the subjacent soil strata (Z_02 and L_02); *Tausonia* under Basidiomycota displayed higher abundance in the subjacent soil strata (Z_02 and L_02), while exhibiting a decline in the surficial mat consortia (Z_01 and L_01). In addition, *Entransia* under Streptophyta displayed higher abundance in Z_01 and L_02, while exhibiting a decline in Z_02 and L_01.

### Environmental variables

**Figure 6:**
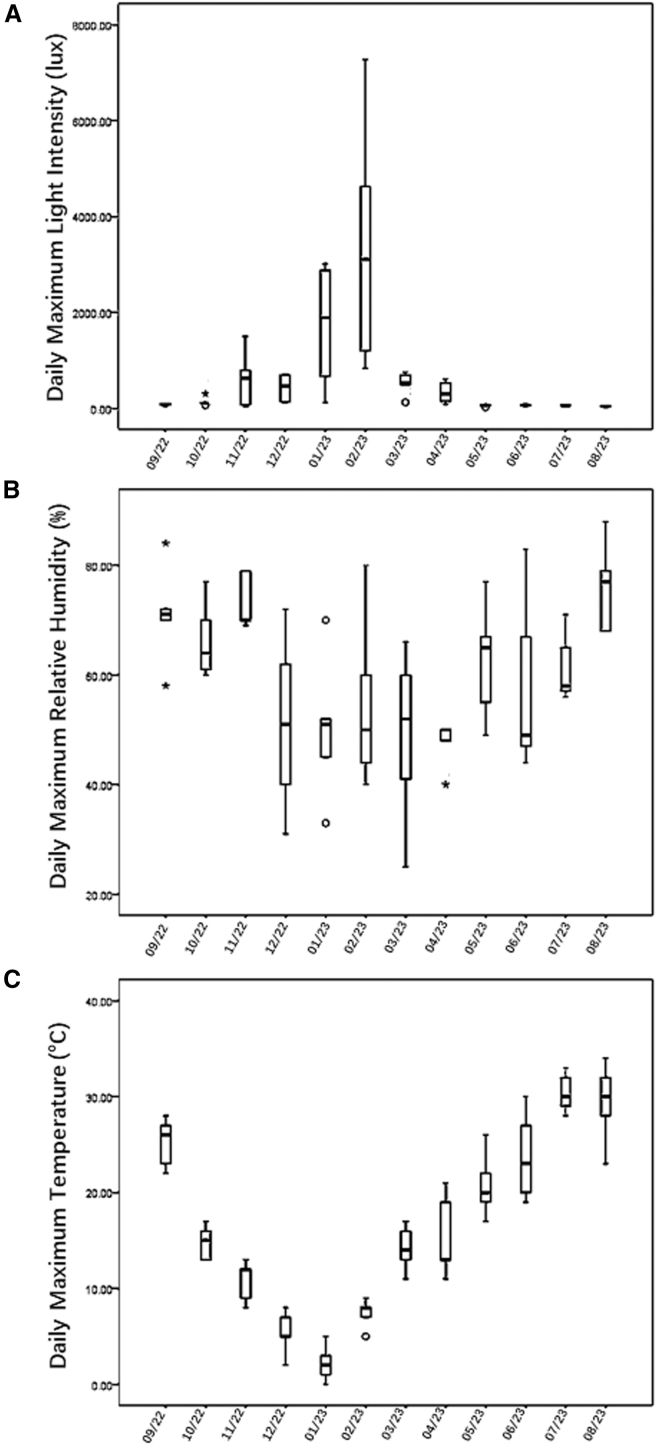
Daily maximum light intensity, relative humidity, and temperature monitored near the purple chromatic anomaly from September 2022 to August 2023 (A) Daily maximum light intensity (Lux) (mean ± SEM: 76.80 ± 11.27, SEP 2022; 139.60 ± 41.09, OCT 2022; 610.00 ± 268.68, NOV 2022; 425.80 ± 130.65, DEC 2022; 1,714.00 ± 578.93, JAN 2023; 3,407.40 ± 1,183.15, FEB 2023; 519.40 ± 110.15, MAR 2023; 335.40 ± 103.61, APR 2023; 55.80 ± 9.13, May 2023; 63.00 ± 9.03, JUN 2023; 54.00 ± 5.02, JUL 2023; 42.80 ± 6.78 AUG 2023). (B) Daily maximum relative humidity (%) (mean ± SEM: 71.00 ± 4.12, SEP 2022; 66.40 ± 3.17, OCT 2022; 73.40 ± 2.29, NOV 2022; 51.20 ± 7.36, DEC 2022; 50.20 ± 6.00, JAN 2023; 54.80 ± 7.14, FEB 2023; 48.80 ± 7.28, MAR 2023; 47.60 ± 1.94, APR 2023; 62.60 ± 4.87, May 2023; 58.00 ± 7.43, JUN 2023; 61.40 ± 2.87, JUL 2023; 76.00 ± 3.76, AUG 2023). (C) Daily maximum temperature (°C) (mean ± SEM: 25.20 ± 1.16, SEP 2022; 14.80 ± 0.80, OCT 2022; 10.80 ± 0.97, NOV 2022; 5.40 ± 1.03, DEC 2022; 2.20 ± 0.86, JAN 2023; 7.40 ± 0.68, FEB 2023; 14.20 ± 1.07, MAR 2023; 15.40 ± 1.94, APR 2023; 20.80 ± 1.53, May 2023; 23.80 ± 2.08, JUN 2023; 30.40 ± 0.93, JUL 2023; 29.40 ± 1.87, AUG 2023) acquired from the real-time microclimate monitoring station deployed within the T5 excavation grid for environmental data collection (September 2022–August 2023).

A multi-parameter environmental sensor array station, comprising light intensity sensors and thermohydrometric loggers, was deployed with a 5-m radius perimeter surrounding the chromatic anomalies, enabling continuous monitoring of intensity of light exposure, ambient temperature, and RH at 30-min intervals across diel cycles (Figure 1B). The chromatic anomalies (purple hue) were predominantly localized within the concave areas of the earthen site, where diffuse sunlight can penetrate through ventilation windows of the protective canopy. We retrieved the environmental data (September 2022–August 2023) acquired from the continuous sensor array proximal to the chromatic anomaly. The data revealed distinct patterns in light intensity during the period from January 2023 to March 2023 (Figure 6A). Post-October 2022, the illuminance parameter began to ascend and, until the period of January–March 2023, remained at a high level of illuminance intensity (600–7,200 lux). After April 2023, the records exhibited a gradual attenuation of illuminance intensity to normal baseline levels in this area (60–100 lux). Continuous monitoring of ambient hygrometric parameters within the chromatic anomaly-affected areas from September 2022 to August 2023 revealed oscillatory RH patterns ranging from 28% to 80%. The frequency distribution pattern demonstrated the higher occurrence of sub-45% RH episodes during December 2022–April 2023, with 42.3% of monitored days exhibiting RH below 45% (Figure 6B). The ambient thermometric surveillance of chromatic anomaly-affected areas during September 2022–August 2023 revealed three phases: autumnal cooling phase (Sep–Oct 2022), with progressive temperature decline observed; winter stabilization phase (Nov 2022–Feb 2023), sustained temperature oscillation between −4°C and 10°C; and spring warming phase (Mar–Aug 2023), with gradual thermal recovery observed (Figure 6C).

## Discussion

In September 2022, during routine conservation monitoring, an unprecedented chromatic anomaly (purple hue) was observed for the first time in the concave areas of the T4 and T5 grids of Pit No. 1 monumental underground chamber. Subsequent conservation monitoring from January 2023 to April 2023 found that the chromatic anomaly had expanded and even spread to the adjacent T24 grid of the archaeological Pit No. 1 monumental site (Figure 1A). Though examination of morphological details in both the chromatic anomaly epicenters and advancing margins within this monumental earthen site, we identified loosely packed microbial mat structures overlying and infiltrating the soil stratum in affected zones (Figures 2C and 2H). By combining morphological and molecular analyses, this study delineates spatial heterogeneity of the microbiome across the chromatic anomaly (purple hue) within Pit No. 1.

At the epicenters of the purple chromatic anomaly, morphological characterization revealed that the filamentous microbes formed complex, vertically stratified networks with distinct structural heterogeneity. SEM analysis showed that the filamentous microbiome—entangled in mat-like networks on soil aggregates—exhibited two distinct morphotypes: (1) stratum-adherent, smooth hyphae exhibiting thigmotropic growth along soil interfaces; and (2) surface-associated hyphae displaying progressive diametric expansion (6–10 μm) and textural roughening, giving rise to a greater diversity of cell morphologies and states of cellular differentiation (Figure 2E). In contrast, the advancing margins of the anomaly exhibited a homogeneous morphological profile (Figure 2I), suggesting an earlier or less differentiated stage of community development.

To characterize the biodiversity underpinning these structural patterns, we performed amplicon sequencing targeting the prokaryotic 16S rRNA gene (V3–V4 region) and the eukaryotic 18S rRNA gene (V4 hypervariable region). This analysis revealed vertical heterogeneity in biodiversity within the purple anomaly epicenter. Specifically, surficial microbial mats (Z_01) exhibited both higher prokaryotic and eukaryotic richness and evenness, whereas the subjacent soil stratum (Z_02) displayed lower diversity. This vertical heterogeneity in biodiversity aligns with the stratified morphological architecture observed by SEM, indicating a structurally and compositionally layered microbiome in the purple anomaly epicenter. In the advancing margin of the chromatic anomaly, however, the biodiversity pattern did not exhibit vertical heterogeneity. For the prokaryotic community, biodiversity metrics revealed greater richness and evenness in the surficial mats (L_01) compared to the subjacent soil stratum (L_02), where both metrics declined. In contrast, the eukaryotic community displayed an opposing pattern: evenness (estimated by the Shannon index) was lower in the surficial mats (L_01) but higher in the subjacent soil stratum (L_02). Although eukaryotic richness in L_01 exceeded that in L_02, the reduced evenness in L_01 indicates the presence of dominant eukaryotic taxa within the surficial mat community (Table S1 in supplemental information). This biodiversity pattern is consistent with morphological observations by SEM. In the advancing margin, the filamentous networks forming the surficial mats consisted predominantly of morphologically filaments with relatively low diversification, characterized by 15–30 μm filamentous frameworks interwoven with smooth, 2- to 5-μm-wide hypha-like filaments on soil aggregates (Figure 2I).

Furthermore, unlike other samples in this study, the taxonomic profiling of the surficial mats from the advancing margin of the chromatic anomaly (L_01) revealed that reads affiliated with Ascomycota alone constituted 87.09% within the eukaryotic consortia in this area. At the genus level, the phylum Ascomycota in this study was primarily composed of *Acarospora* and *Aspergillus*. *Acarospora* is a distinct genus of predominantly crustose lichenized fungi, growing on exposed rocks and soil on all continents.^12^ *Aspergillus*, a filamentous fungal genus, has high adaptability to survive within a broad range of environmental conditions. *Aspergillus* is able to metabolize not only simple sugars but also a wide range of substrates such as polysaccharides, proteins, or other large organic molecules, therefore growing well in soil, decaying vegetation, hay, dung, compost piles, hospital air-conditioner filters, and potted plants, and can even be isolated from the air.^13^ Pit No. 1 of the Terracotta Army monumental complex is a large-scale semi-exposed earthen site featuring a protective canopy structure with circumferential ventilation windows that enable air exchange between the archaeological matrix and external atmospheric conditions.^2^ Concurrently, the peripheral visitor circulation corridor sustains substantial anthropogenic loading.^3^ The exogenous atmospheric influx together with intensive anthropogenic activities facilitated the continuous transport and subsequent deposition of bioaerosol-borne particulates, including botanical trichomes, cellulose-based textile fibers, spore-laden dust aggregates, and microbial propagules, onto the earthen substrate surfaces of Pit 1.^14^ The absorption and deposition of bioaerosol-borne particulates onto the surface of the earthen heritage substrates facilitate the retention of bioavailable organic substrates, thereby rendering this earthen site susceptible to microbial colonization and subsequent mat-like microbial succession. When the environmental condition is suitable, organic matter-degrading microorganisms within the soil stratum initiate colonization.^14^ Given the predominantly heterotrophic nature of the Ascomycota taxa, which is abundant in the surficial mats from advancing margin of the chromatic anomaly (L_01), the observed spatial heterogeneity patterns of eukaryotic consortia across chromatic anomaly (purple hue) within Pit No. 1 reflected the microhabitats for the growth of microorganisms that are created when aggregation of flocs promotes the adsorption of eukaryotic microbes on the surface of the earthen site and when the micro-climate is suitable for their resuscitation.

Cyanobacteria were the most abundant prokaryotic phylum in both surficial mat samples (Z_01 and L_01) (Figure 4A). As the primary phototrophic component of the Pit No. 1 microbiome, this autotrophic guild not only provides the organic carbon that drives the development and ecological succession of the microbial community within the chromatic anomaly but also structures the surficial mat architecture. The cyanobacterial taxa associated with *Iphinoe* LOS-B1 and EcFYyy-200 together demonstrated high relative abundance in prokaryotic consortia within the surficial mats in this study. These cyanobacterial taxa collectively constitute in excess of 60% of the prokaryotic consortia in superficial mats (Z_01 and L_01), with relative abundance peaking at 70% in specimen Z_01 collected from the purple anomaly epicenter (Figure 4B). Unlike the generalist cyanobacteria that can tolerate broad environmental fluctuations, the *Iphinoe* LOS-B1-related and *Oscillatoriales* EcFYyy-200-related cyanobacterial clades thrive typically under specific troglophilic conditions and exhibit specific microclimatic requirements. These taxa are previously documented as specialists in subterranean habitats like karst caves and hypogea and exhibit distinct troglophilic phenotypic and ecophysiological characteristics.^6^^,^^8^^,^^9^^,^^15^ For example, the presence of the *Iphinoe* LOS-B1-related cyanobacterial clade and the *Oscillatoriales* cyanobacterium associated with the EcFYyy-200 clade in microflora has been reported in geographically isolated karstic cave ecosystems in the Mediterranean area, such as Greece and Spain,^8^^,^^9^ and in subterranean crypts, catacombs, and hypogea in Malta, Italy, and Spain,^6^^,^^15^ respectively. The red filamentous cyanobacterial members belonging to *Oscillatoriales* are commonly distributed in hypogea with fine, thin purple-red trichomes (∼3.5 μm in width). The microbes possessed morphological characters forming mat-like thalli and ultimately reddish coatings over calcareous substrata. Their patchy biofilms colonized limestone, plaster, mortar, stuccoes, and wall paintings in specific environments, such as subterranean crypts, catacombs, and hypogea.^6^^,^^16^ The *Iphinoe* LOS-B1-related cyanobacterial clade has been widely reported as a resident of cave systems, occurring in various subterranean habitats including hollows, cavities, and grottos.^6^^,^^15^ Its thallus is partially endolithic and creeps on calcareous substrates at the cave illuminated zone as a whitish-silver to purple coating, occasionally interwoven with other cyanobacteria. The trichomes are predominantly cylindrical, slightly constricted at cross-walls or torulose, and enclosed within non-pigmented sheaths; the cytoplasm appears violet. Trichome breakage and release of hormocysts occur by enlargement of sheaths between neighboring cells.^8^ The genus *Iphinoe* is characterized by obligatory true branching, in addition to reproduction by hormocysts.^8^^,^^9^ In our specimens collected from the purple anomaly epicenter (e.g., Z_), the morphological traits observed under LM closely correspond to the aforementioned description (Figure 2D). The surface-adherent microbial filaments within the filamentous mat were uniseriate and ensheathed in a colorless mucilaginous layer, comprising cylindrical to torulose cells measuring 5–7 μm in width and 6–10 μm in length, and characterized by magenta-violet granulated cytoplasm. These filaments formed a loose, mat-like architecture with frequent branching, and branchpoint cells were discernible. Furthermore, hormocyst release was observed to occur through sheath enlargement between neighboring cells; these hormocysts were occasionally formed at the ends of the main filaments (Figure 2D). Owing to true branching, filaments exhibited variable orientations on the mat surface, forming a three-dimensional scaffold. This true-branching habit, together with hormocyst reproduction, explains the observed three-dimensional filamentous network and the progressive hyphal inflation (diameter gradient 6–10 μm) with rough textures indicative of cellular differentiation in the epicenter mats (Z_) (Figure 2E). These phenotypic traits closely match those reported for the *Iphinoe* clade.

Although subterranean habitats—such as caves and hypogea—represent confined and unique biotopes characterized by stable RH, cool air temperatures, and, crucially, limited light intensity that constrains photosynthetic life, they serve as refugia for specialized cyanobacterial clades. These microorganisms are well adapted to survive under specific light conditions and have developed distinct troglophilic ecological and morphological traits. Both the *Iphinoe* LOS-B1-related cyanobacterial clade and the *Oscillatoriales* cyanobacterium associated with the EcFYyy-200 clade are components of natural populations inhabiting these highly specialized environments. They exhibit a disjointed but worldwide distribution in subterranean environments, where dim light seems to be a major stress factor. Despite generally low-light conditions in subterranean environments, phototrophic microhabitats can develop where sufficient natural light penetrates or where artificial lighting is present.^8^ For example, the *Iphinoe* LOS-B1-related cyanobacterial clade has been isolated from biofilms occurring both at cave entrances where natural light penetrates and in transitional zones equipped with artificial lighting. Zammit reported that *Oscillatoriales* troglophilic cyanobacterial isolates from catacombs in Rome were maintained on agar-solidified BG11 medium with a light intensity of 10 μmol photons m^−2^ s^−1^ (∼556 lux) provided by cool-white fluorescent tubes where growth was observed.^6^

The morphological and taxonomic consistency supports the identification of the colonizers responsible for the purple chromatic anomaly in Pit No. 1 as troglophilic specialists, whose niche preferences can be reflected in the microclimatic conditions prevailing during the outbreak period. In this preliminary study, the morphological and molecular similarity to previously reported troglophilic specialist cyanobacteria can be attributed to specific microenvironmental parameters during the outbreak period. Similarities can be drawn between the environmental parameters of these subterranean sites where these troglophilic specialist cyanobacteria were found and the spatiotemporally heterogeneous microclimatic parameters observed in the specific concave areas of Pit No. 1 where the chromatic anomaly emerged. Before April 2023, the purple anomalous area was found to have rapidly expanded, which can be considered a niche invasion event. These specialized cave-adapted cyanobacteria (e.g., *Iphinoe*-related clades and the *Oscillatoriales*-related EcFYyy-200 clade) may have been introduced into Pit No.1 via exogenous influx in September 2022. However, an outbreak requires specific microclimatic conditions. Environmental monitoring from January 2023 to March 2023 revealed a defining period characterized by three key factors: elevated illuminance (peaking at 600–7200 lux between January and March), frequent low-humidity episodes (RH < 45% on 42.3% of days from December to April), and a stable low-temperature phase oscillating between −4°C and 10°C (November to February) (Figure 6). During this period, the transient environmental regime, which combined high light intensity, periodic low humidity, and cool but stable temperatures, had created an empty niche—a photic, subterranean structural surface on earthen substrates—that was exploited by these pre-adapted troglophilic cyanobacteria.

This study integrated morphological, molecular, and microclimatic analyses and revealed that the purple chromatic anomaly was linked to specialized troglophilic cyanobacteria (*Iphinoe* LOS-B1-related clade and the *Oscillatoriales* cyanobacterium associated with the EcFYyy-200 clade), which typically inhabit calcareous subterranean environments but have successfully colonized earthen substrates under a specific microclimatic regime. Microclimatic monitoring identified the environmental triggers: a combination of high light exposure (peaking at 600–7,200 lux between January and March 2023), periodic low humidity (RH < 45% during 42.3% of days from December to April), and stable cool temperatures (−4°C to 10°C from November to February). This unique set of conditions created an empty ecological niche that facilitated the rapid expansion of pre-adapted troglophile specialists within the microbial consortium. These results underscore that even within semi-enclosed heritage environments, localized microclimatic variations can induce microbial outbreaks capable of altering the aesthetic integrity of earthen substrates in the monumental underground heritage site. Subsequent in-depth conservation research will systematically employ sampling strategies using high-density sensor array stations spanning both the epicenter and peripheral zones. These future studies will also incorporate more detailed microclimatic and pedo-geochemical variables—such as soil moisture, porosity, mineral composition, trace elements, light intensity, and soil temperature—to precisely quantify the correlation and combined effects of specific drivers on this microbial coloration.

### Limitations of the study

There are several limitations in this study. First, as a preliminary investigation conducted on a UNESCO World Heritage site, sampling was strictly minimized to four specimens within two spatial loci (epicenter Z_ and advancing margin L_) to avoid any aesthetic or structural damage to the irreplaceable earthen heritage. Consequently, the sample size is limited, and biological replicates were not included, which may affect the generalizability of the diversity and taxonomic findings. Second, the environmental monitoring sensor array was deployed after the initial detection of the purple anomaly (September 2022), and only a single station was placed adjacent to the affected area. Therefore, direct comparative illuminance measurements between the epicenter (Z_) and the advancing margin (L_) were not available, and historical light data prior to September 2022 could not be obtained. This restricts our ability to determine whether the observed microclimatic regime was truly unprecedented or had occurred previously at this site. Finally, this study focused on the microbial community composition and microclimatic drivers, but pedo-geochemical variables (e.g., soil moisture, porosity, mineral composition, and trace elements) were not analyzed. Future in-depth studies should incorporate high-density sensor arrays, systematic sampling designs with biological replicates, and comprehensive geochemical characterization to precisely quantify the combined effects of multiple drivers on the purple chromatic anomaly.

## Resource availability

### Lead contact

Further information and requests for resources should be directed to and will be fulfilled by the lead contact, Xinghua Ding (diaos37@hotmail.com).

### Materials availability

This study did not generate new unique reagents.

### Data and code availability

• Amplicon high-throughput sequencing data have been deposited at Sequence Read Archive (SRA) in NCBI as BioProject PRJNA1345248 under accession number SUB15708499 (SRA: PRJNA1345248), and other datasets are available from the corresponding author on request.
• This study did not generate code.
• Any additional information required to reanalyze the data reported in this paper is available from the lead contact upon request.

## Acknowledgments

The work was supported by “Research on the Mechanism of the Occurrence of ‘Purple’ Microbial Diseases on the Surface of the Qin Terracotta Army Pit No. 1 Site” (project no. 2023ZCK025), The 2023 National Cultural Relics Administration’s Scientific and Technological Project for Cultural Relics. The study was financially supported by the 10.13039/501100015401Key Research and Development Program of Shaanxi (project no. 2022LL-ZD-01), the Key R&D Program Project of Shaanxi Province (project no. 2021ZDLSF-06-01), and the Open Fund of Hunan Provincial Key Laboratory of Intelligent Protection and Utilization of Brick and Stone Cultural Relics, Hunan University of Science and Engineering (project no. HUSE-2024-07).

## Author contributions

Q.L.: conceptualization, data curation, methodology, supervision, investigation, and writing – original draft; Z.Y.: investigation, methodology, and writing – review and editing; P.Z.: writing – review and editing, formal analysis, and funding acquisition; F.W.: writing – review and editing; X.M.: writing – review and editing; D.H.: data curation and writing – review and editing; N.X.: data curation and writing – review and editing; J.L.: data curation and writing – review and editing; Y.X.: writing – review and editing; H.L.: data curation; M.S.: writing – review and editing; X.D.: conceptualization, data curation, investigation, methodology, supervision, and writing – review and editing. All authors have read and agreed to the published version of the manuscript.

## Declaration of interests

The authors declare no competing interests.

## STAR★Methods

### Key resources table

**Table:** 

REAGENT or RESOURCE	SOURCE	IDENTIFIER
**Biological samples**		

Microbial mat samples	This paper	NA
Crust soil samples	This paper	NA

**Chemicals, peptides, and recombinant proteins**		

TransStart® Fast pfu DNA Polymerase (with 2.5 mM dNTPs)	TransGen Biotech Co., Ltd	Cat# AP221-11
Phusion HF mix	Thermo Fisher Scientific Inc.	Cat# F531L
DMSO	Thermo Fisher Scientific Inc.	Cat# F515
Vazyme VAHTSTM DNA Clean Beads	Vazyme Biotech Co., Ltd.	Cat# N411

**Critical commercial assays**		

Quant-iT Pico Green dsDNA Assay Kit	Thermo Fisher Scientific Inc.	Cat# P11496
NovaSeq 6000 SP Reagent Kit (500 cycles)	Illumina	Cat# 20028402

**Deposited data**		

Amplicon High-Throughput Sequencing data reported in this study	Sequence Read Archive (SRA) in NCBI	SUB15708499; SRA: PRJNA1345248

**Oligonucleotides**		

Primer 16S rRNA 338F Forward: ACTCCTACGGGAGGCAGCA;	This Paper	NA
Primer 16S rRNA 806R Reverse: GGACTACHVGGGTWTCTAAT	This Paper	NA
Primer 18S rRNA TAReuk454FWD1 Forward: CCAGCASCYGCGGTAATTCC	This Paper	NA
Primer 18S rRNA TAReukREV3 Reverse: ACTTTCGTTCTTGATYRA	This Paper	NA

**Software and algorithms**		

QIIME2	Ewels et al.^17^	https://gregcaporaso.github.io/fq-manifestor-web/
qiime cutadapt trim-paired command v.2025.4.0	Martin et al.^18^	https://github.com/qiime2/q2-cutadapt
QIIME2 feature-classifier plugin v.2025.4.0	Bokulich et al.^19^	https://github.com/qiime2/q2-feature-classifier
R package ‘‘dada2’’ v.1.26.0	Callahan et al.^20^	https://github.com/benjjneb/dada2
SILVA Release 132 database	Quast et al.^21^	https://www.arb-silva.de/documentation/release-132/
MAFFT v.7.487	Katoh^22^	https://mafft.cbrc.jp/alignment/software/
FastTree v.2.2.0	Price et al.^23^	https://github.com/morgannprice/fasttree
qiime feature-table rarefy command v.2025.7.0	Weiss et al.^24^	https://github.com/qiime2/q2-feature-table
MEGEN v.6.25.10	Huson et al.^25^	https://github.com/husonlab/megan-ce
R package ‘‘VennDiagram’’ v.1.8.2	Zaura et al.^26^	https://github.com/uclahs-cds/package-VennDiagram

**Other**		

HOBO® Light Intensity Data Logger (HLI)	Onset Computer Corporation	Cat# 470330-995
iButton® temperature/humidity logger	Maxim Integrated Products, Inc.	Cat# DS1923-F5

### Experimental model and study participant details

Omitted as our study does not involve biological models.

This study did not involve the use of experimental models (animals, human participants, plants, microbial strains, cell lines, or primary cell cultures).

### Method details

#### Sampling site and environmental data acquisition

Pit No. 1 of the Terracotta Army (34°23′06″ N; 109°16′23″ E; altitude 460 m a.s.l.) is a renowned heritage site near Lintong District, located on the eastern side of Mount Li (Shaanxi, China). Geographically, this site located in a warm-temperate semi-humid continental monsoon climate prevails with an annual precipitation of 558.5 mm, of which more than 240 mm is concentrated mainly in three months from July to September. The annual relative humidity is 69%, and the annual temperature is 13.5°C, varying over a range from −6.5°C to 41.7°C. These surrounding climate variables obtained from the World Weather Online database (https://www.ncdc.noaa.gov/cdo-web). The Pit No. 1 site is a monumental underground chamber and the pit structure measures approximately 230 m in length, 62 m in width, and covers a total area of 14,260 square meters. It contains more than 6,000 life-sized terracotta figures, primarily arranged in military formation, with the majority being infantry and chariots positioned in orderly corridors. The excavation mouth ranges from 0.3 to 1.5 m in elevation above the present ground level, with its base extending to depths of 4.5–6.5 m below the excavation mouth.^11^

In September 2022, during routine conservation monitoring, an unprecedented chromatic anomaly (purple hue) was observed for the first time on the specific concave areas of the T4 and T5 grids of Pit No. 1 monumental underground chamber. Routine conservation monitoring revealed that the purple anomaly had expanded rapidly from January 2023 to April 2023 and had also appeared on the side walls of the adjacent T24 grid. The T5 grid, which was the initial site where the purple contamination appeared, was selected for sample collection and microbial analyses during the March 2023. After the April 2023, the continuously monitoring observation found that the purple contamination shows no signs of expansion and has stabilized. And in September 2022, following the discovery of this unprecedented purple chromatic anomaly, a multi-parameter environmental sensor array station (light intensity sensors and thermohydrometric loggers) was deployed within a 5-meter radius around the anomaly within the T5 grid—the initial emergence area—to characterize the proximal microclimate. The a multi-parameter environmental sensor array station, comprising light intensity sensors and thermohydrometric loggers, enabling continuous monitoring of intensity of light exposure, ambient temperature, and relative humidity at 30-min intervals across diel cycles (Figure 1B). The light intensity (LI) data were recorded every 30-min for a year using a HOBO Light Intensity Data logger; temperature (TEMP., ◦C) and relative humidity (RH, %) were recorded every 30-min for a year via the iButton temperature/humidity logger (DS1923). The environmental data acquired from this environmental continuous sensor array proximal to chromatic anomaly (purple hue) were retrieved from September 2022 to August 2023.

#### Sample collection

Given the uniqueness and irreplaceability of the UNESCO World Heritage Site, a preliminary study is typically required to establish baseline data on the microbiome to guide subsequent in-depth conservation research. Accordingly, sampling in this preliminary investigation was authorized under the strict requirement that it be minimized to four sampling sites within two loci (Z_: the epicenter area; L_: the advancing margin area), with surfaces of aesthetic visibility anywhere in Pit No. 1 avoided. Two of the sampling sites were designated for morphological and microscopic analysis, while the other two were allocated for molecular biological analysis.

For morphological analysis sampling, the sampling strategy was designed to capture heterogeneity across the chromatic anomaly, with *in situ* collection conducted at two distinct loci: the purple chromatic anomaly hotspots (epicenter zones, designated Z_) and the advancing margins (peripheral zones, designated L_). Crust samples with microbial mats and microbe-infiltrated interfaces, were collected from both the epicenter zones and advancing margins within the T5 grid—the area where the purple contamination initially emerged—to characterize their micromorphological features using microscopic techniques. Sampling points for morphological and microscopic analysis were located on the side walls of this monumental chamber to avoid disturbing any visually exposed surfaces to visitors within the Pit No. 1 site. Approximately 6 g of material were collected from each sampling location within the purple anomaly-affected zones of the T5 grid using sterile spatulas and aseptic forceps.

For molecular analysis, four specimen subtypes were collected from two spatial loci (the purple epicenter and the advancing margin). In the epicenter area of the purple anomaly, approximately 2 g of surficial loosely packed microbial mat (designated Z_01) was first collected from a 2 cm × 2 cm area, followed by approximately 2 g of the underlying earthen substrate (designated Z_02) from the same 2 cm × 2 cm area at a depth of 0.5–1.0 cm beneath the mat. In the advancing margin area of the purple anomaly, approximately 2 g of surficial loosely packed microbial mat (designated L_01) was first collected from a 2 cm × 2 cm area, followed by approximately 2 g of the underlying earthen substrate (designated L_02) from the same 2 cm × 2 cm area at a depth of 0.5–1.0 cm beneath the mat. After collection, the samples were placed in an ice-box for immediate storage and then sent to the laboratory for further analysis within 48 h. To ensure no compromise to the site’s aesthetics, sampling was restricted to non-visible areas of the pit walls, leaving the visual appearance of the Terracotta Army Pit No. 1 unimpaired. The sampling procedure was performed on March 2023 under the authorization and supervision of the Cultural Relics Preservation Department of Emperor Qinshihuang’s Mausoleum Site Museum.

#### Microscopic morphology observation

Field-based observation of the surface topology was performed across the purple chromatic anomaly affecting zone in T5 grid by using a Anyty 3R-MSA200WF handheld digital microscope (200× magnification), with particular focus on both the chromatic anomaly epicenters (exhibiting purple hue) and their advancing marginal zones to document the spatial pattern of surface topology and visual characteristics of the chromatic anomaly (Figures 2A, 2C, 2F, and 2H).

And then, the ultra-depth of field observation was conducted for obtaining microscopic morphology characteristics of the crust samples with microbial-infiltrated interface from the purple anomalous affecting zones (Figures 2B and 2G). For light microscopy (LM), the loosely packed microbial mat collected from the purple patches was observed on glass slides under a high-resolution light microscope (DM 2700M; LEICA) (Figure 2D). The advanced morphology characterizations were examined using a **S**canning **E**lectron **M**icroscope (FEI QUANTA 650). The undamaged fragments of the specimens were affixed onto a brass specimen holder using double-sided adhesive tape stuck on a vacuum-clean stub, and gold spraying was treated on samples twice for 60 s under vacuum prior to observation. SEM working parameters were as follows: accelerating voltage of 10 KV, electrofocusing of 25 mm, current probe of 1 Na, and spectral collection time of 60–300 s (Figures 2E and 2I).

#### High-throughput amplicon sequencing

Total genomic DNA from the microbial mat samples (Z_01 & L_01) were extracted by using the OMEGA Soil DNA Kit (M5635-02) (Omega Bio-Tek, Norcross, GA, USA) according to the manufacturer’s protocols. For each sample, the integrity of extracted DNA was determined by electrophoresis in 1.0% agarose gels, and the purity and concentration of the extracted DNA were measured spectrophotometrically with a NanoDrop ND-2000 (Thermo Fisher Scientific, Waltham, MA, USA). The universal primers 338**F**/806**R** (F: ACTCCTACGGGAGGCAGCA; R: GGACTACHVGGGTWTCTAAT) targeting prokaryotic 16S rRNA V3-V4 hypervariable regions^27^ and TAReuk454**F**WD1/TAReuk**R**EV3 primers (F: CCAGCASCYGCGGTAATTCC; R: ACTTTCGTTCTTGATYRA) targeting eukaryotic 18S rRNA V4 loci^28^ were used for 1st-step PCR amplification. The 1st-step PCR was performed in a 25 μL total volume. The PCR components contained 5 μL of buffer (5×), 0.25 μL of Fast pfu DNA Polymerase (5U/μL), 2 μL (2.5 mM) of dNTPs, 1 μL (10 μM) of each Forward and Reverse primer, 1 μL of DNA Template, and 14.75 μL of ddH2O. PCR for prokaryotic 16S rRNA V3-V4 hypervariable regions was performed as follows: initial denaturation at 98°C for 5 min, followed by 25 cycles consisting of denaturation at 98°C for 30 s, annealing at 53°C for 30 s, and extension at 72°C for 45 s, with a final extension of 5 min at 72°C; PCR for eukaryotic 18S rRNA V4 loci was performed as follows: an initial denaturation step at 95°C for 2 min, then 24 cycles of 95°C for 30 s, 52°C for 30 s, and 72°C for 30 s; followed by a final extension at 72°C for 5 min.

Sample-specific 7-bp barcodes enabling multiplexing were added in the 2^nd^-step PCR. For this, a 100 μL PCR mixture was prepared using 10 μL of the 1st-step PCR product, 1ⅹPhusion HF buffer, 0.2 mM dNTPs, 0.125 μM each forward and reverse barcode primer, 0.25% DMSO, and 0.5 μL of Phusion HF II DNA polymerase. PCR conditions were 98°C for 40 s, 10 cycles of 98°C for 20 s, 55°C for 40 s, and 72°C for 40 s, and a final extension step at 72°C for 2min. Further details and work time estimations are found in the work of.^29^ For validation and quality assurance, 8 μL of the 2nd-step PCR product was loaded onto a 1.5% agarose gel. The remaining 92 μL of the 2nd-step PCR products were purified with Vazyme VAHTSTM DNA Clean Beads (Vazyme, Nanjing, China). Concentrations of the final PCR products were measured using the Quant-iT PicoGreen dsDNA Assay Kit (Invitrogen, Carlsbad, CA, USA). After the individual quantification step, amplicons were pooled in equal amounts, and pair-end 2 × 250 bp sequencing was performed using the Illlumina NovaSeq platform with NovaSeq 6000 SP Reagent Kit (500 cycles).

### Quantification and statistical analysis

#### Processing and analysis of the sequencing data

The QIIME2 (2019.4) was employed for primer removal, quality filtering, denoising, read merging, and chimera removal of the raw sequencing read data.^17^ The denoising procedure was implemented in QIIME2 (2019.4). First, primer sequences were trimmed using the qiime cutadapt trim-paired command, and sequences failing to match the expected primers were discarded.^18^ Subsequently, quality control, denoising, merging, and chimera removal were performed using the qiime dada2 denoise-paired command, which invokes the DADA2 algorithm.^20^ Unlike traditional OTU-picking methods that cluster sequences at a similarity threshold, DADA2 performs dereplication (equivalent to clustering at 100% similarity) to infer biological sequence variants. The dereplicated, denoised sequences generated through this process are referred to as amplicon sequence variants (ASVs). Following denoising, ASV feature sequences and the corresponding ASV table were merged, and singleton ASVs (i.e., ASVs with a total abundance of one across all samples) were removed as a default filtering step.

Illumina NovaSeq 6000 2 × 250 bp paired-end sequencing yielded a total of 399,051 raw reads from the 16S prokaryotic libraries across the four molecular samples. After primer trimming (cutadapt), quality filtering, and denoising (DADA2 with default parameters except truncLen = 240 for forward reads and 200 for reverse reads, maxEE = 2), a total of 335,817 high-quality merged reads remained. For the 18S eukaryotic libraries, a total of 345,752 raw reads were obtained from the same four samples. After the same trimming, filtering, and denoising steps, 327,435 high-quality merged reads remained. These were assigned to 280,963 non-singleton ASVs’ reads (ASVs abundance) for the prokaryotic dataset and to 314,846 non-singleton ASVs’ reads for the eukaryotic dataset. Per-sample sequencing depths (ASVs abundance) ranged from 45,557 to 83,500 reads (mean = 70,241) for the 16S libraries and from 66,628 to 90,236 reads (mean = 78,712) for the 18S libraries (Table S2).

Taxonomic annotation of ASVs was performed by aligning representative sequences against reference databases using a naive Bayes taxonomy classifier. The annotated Amplicon Sequence Variants (ASVs) for each taxonomic group are summarized in Table S3. Specifically, the classify-sklearn algorithm within the QIIME2 feature-classifier plugin^19^ was employed with default parameters against the SILVA Release 132 database.^21^ It should be noted that due to the vast diversity of microorganisms, reference databases remain incomplete, leading to some sequences being labeled as “unassigned” or lacking precise taxonomic resolution (e.g., “unidentified,” “uncultured,” “incertae sedis”). Additionally, sequencing read length constraints may preclude genus- or species-level classification for certain ASVs, resulting in annotations at higher taxonomic ranks only. To infer phylogenetic relationships and genetic distances among ASVs, a phylogenetic tree was constructed. Multiple sequence alignment was performed using MAFFT^22^ via the qiime phylogeny align-to-tree-mafft-fasttree pipeline in QIIME2. Regions lacking phylogenetic information were masked, and a maximum-likelihood tree was built using FastTree,^23^ generating both an unrooted and a midpoint-rooted tree.

To standardize sequencing depth across samples for downstream comparative analyses, rarefaction was performed. This approach randomly subsamples reads from each sample to a uniform depth, enabling estimation of ASV richness and relative abundances at a standardized sequencing effort.^30^^,^^31^ Rarefaction was implemented in QIIME2 using the qiime feature-table rarefy command, with the sampling depth set to 95% of the minimum sequence count observed across all samples. The taxonomy compositions and abundances were visualized using MEGAN.^25^ Venn diagram was generated to visualize the shared and unique ASVs among samples using R package “VennDiagram”, based on the occurrence of ASVs across samples regardless of their relative abundance.^26^ Hierarchical clustering analysis (heatmap) using the average linkage method was performed on the rarefied feature table using the Bray-Curtis distance metric. Rows represent how samples relate by community composition, as arranged by the horizontal dendrogram. Clustering was performed on the rarefied feature table summarized to taxonomy annotation of genus level, but only the top 30 genus of the total microbiome are displayed. The raw sequences were deposited in the NCBI Sequence Read Archive (SRA Database) as BioProject PRJNA1345248 under accession number SUB15708499.
