# Supplementary material for: Microbial succession and microclimatic fluctuation in purple chromatic anomaly on Terracotta Warriors Pit 1
Source: iScience. 2026 Jul 10;29(8):116717. doi: 10.1016/j.isci.2026.116717 (PMC13380436; doi:10.1016/j.isci.2026.116717)
Supplement: Document S1. Figures S1 and S2 and Tables S1–S3 [file mmc1.pdf]

iScience, Volume *29*

Supplemental information

Microbial succession and microclimatic fluctuation in purple chromatic anomaly on Terracotta Warriors Pit 1

Qiang Luo, Zinan Yang, Ping Zhou, Fasi Wu, Xiaofen Mao, Dongpeng He, Na Xi, Jie Li, Yin Xia, Hua Li, Maosheng Shen, and Xinghua Ding

Supplementary materials

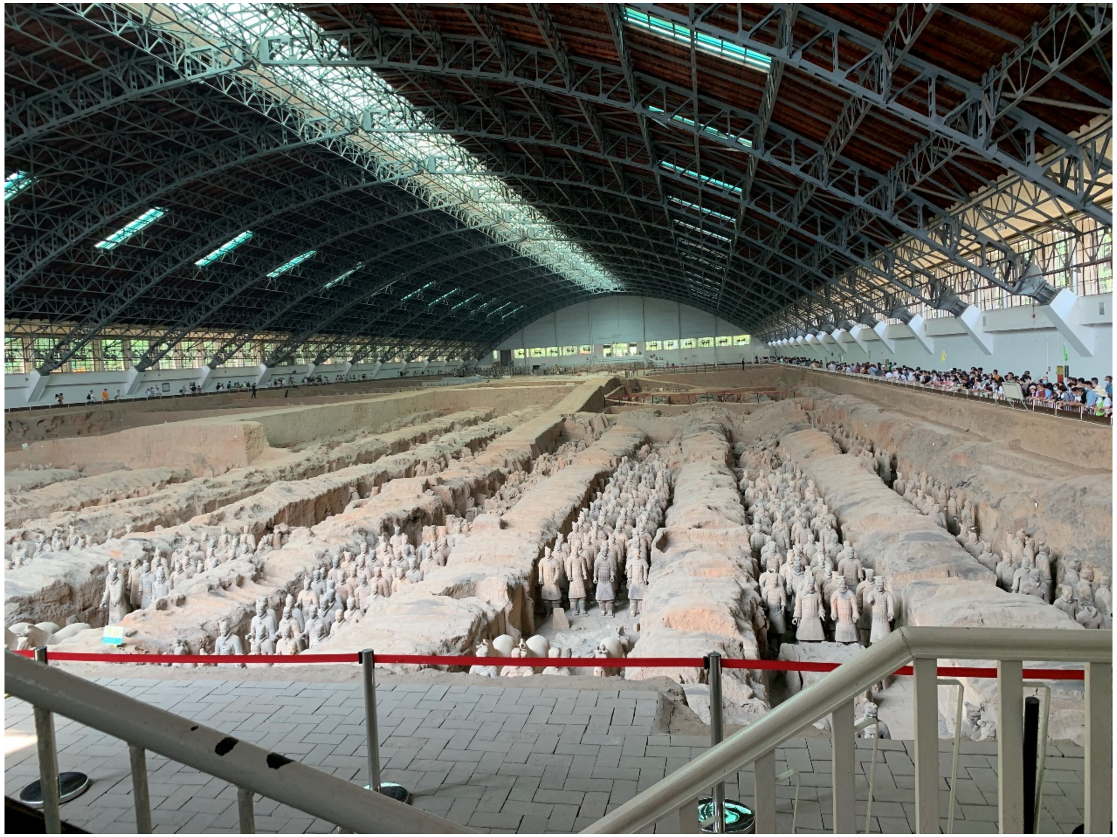

Figure S1. Protection shed and exhibition hall over the monumental underground chamber of the Terracotta Warriors Pit No.1

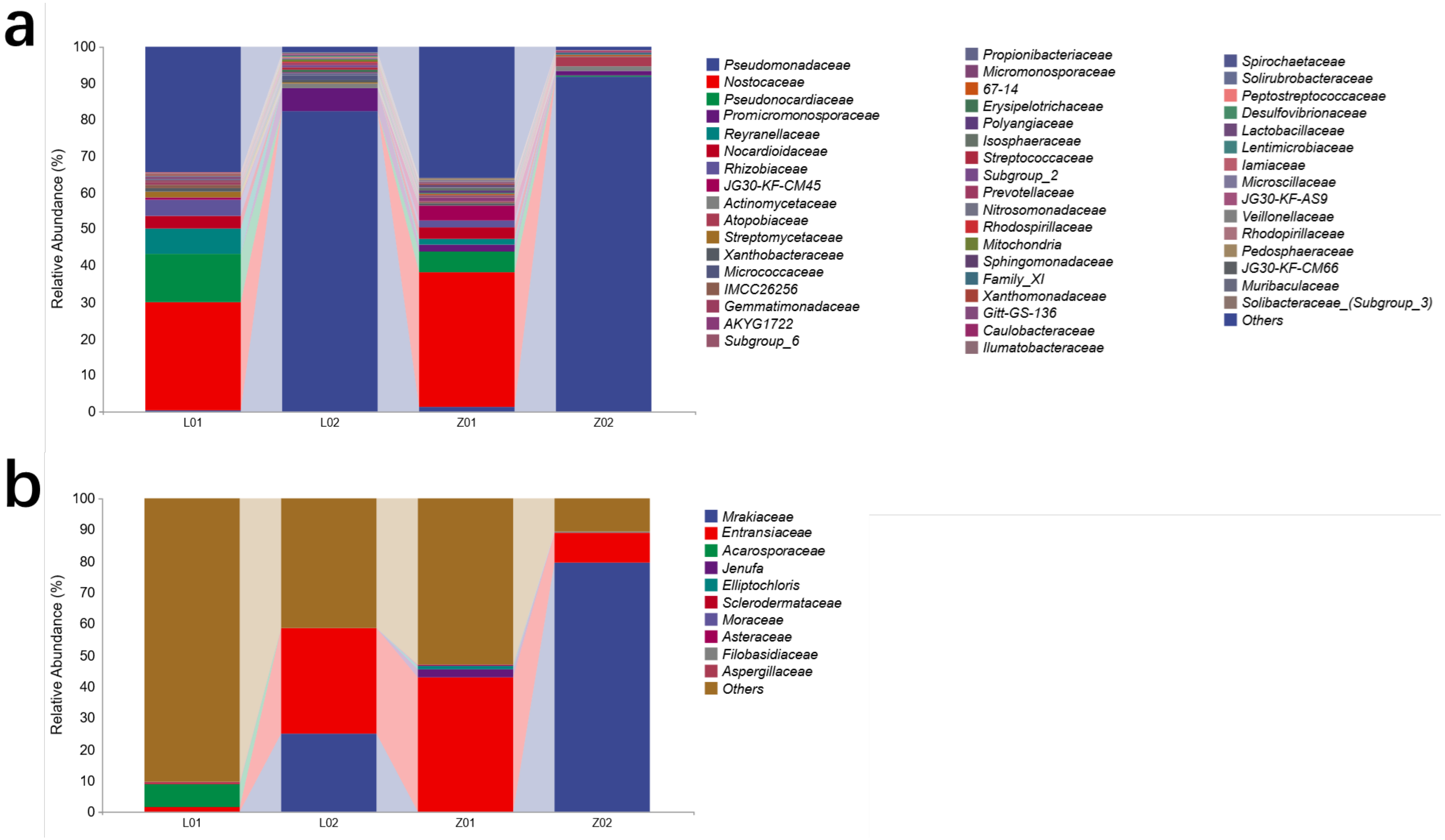

Figure S2. Family-level abundance distribution of prokaryotic and eukaryotic consortia across purple chromatic anomaly zones.

a) Abundance distribution of prokaryotic consortia at family level across both chromatic anomaly (purple hue) core zone and marginal zone; **b)** Abundance distribution of eukaryotic consortia at family level across both chromatic anomaly (purple hue) core zone and marginal zone. (Z01 & L01——samples of surficial mats across the chromatic anomaly epicenters and advancing marginal zones; Z02 & L02——samples of the subjacent soil strata across the chromatic anomaly epicenters and advancing marginal zones)

Table S1. Microbial diversity indices of prokaryotic and eukaryotic consortia in surficial mats and subjacent soil strata across purple anomaly epicenter and advancing margin.

(a) Prokaryotic diversity characteristics; **(b)** Eukaryotic diversity characteristics.

a

SAMPLE	CHAO1	OBSERVED_ASVS	SHANNON	SIMPSON
L01	472. 76	431	3. 93	0. 82
L02	376. 62	353	2. 04	0. 38
Z01	1338. 94	1336	4. 65	0. 80
Z02	205. 53	190	1. 01	0. 19

b

SAMPLE	CHAO1	OBSERVED_ASVS	SHANNON	SIMPSON
L01	84. 87	84	1. 05	0. 27
L02	47	47	1. 99	0. 69
Z01	217. 18	214	2. 42	0. 65
Z02	24. 1	24	1. 2	0. 36

Table S2. Summary of sequencing data processing and read depths for 16S and 18S libraries.

(a) 16S prokaryotic libraries. (b) 18S eukaryotic libraries

a

SampleID	Input	Filtered	Denoised	Merged	Non chimeric	Non singleton
**Z01**	91036	84494	81372	73946	46416	45557
**Z02**	105397	89024	88264	87560	83530	83500
**L01**	103611	97081	96062	93784	78850	78687
**L02**	99007	82946	82077	80527	73317	73219
**Total**	399051	353545	347775	335817	282113	280963

b

SampleID	Input	Filtered	Denoised	Merged	Non chimeric	Non singleton
**Z01**	101570	96590	96394	95926	90252	90236
**Z02**	71110	67881	67859	67777	66628	66628
**L01**	91470	86826	86696	86455	83141	83138
**L02**	81602	77520	77381	77277	74844	74844
**Total**	345752	328817	328330	327435	314865	314846

Table S3. Number of taxonomically annotated ASV variants for prokaryotic and eukaryotic datasets.

(a) prokaryotic ASVs and (b) eukaryotic ASV Variants.

a

ID	phylum	class	order	family	genus	species
**Z01**	11	53	38	179	940	86
**Z02**	2	1	9	9	133	32
**L01**	2	21	23	75	290	13
**L02**	2	13	10	25	231	62
**Total**	17	88	80	288	1594	193

b

ID	phylum	class	order	family	genus	species
**Z01**	44	55	10	11	25	62
**Z02**	4	2	0	0	4	11
**L01**	1	12	32	11	14	10
**L02**	7	7	1	1	3	23
**Total**	56	76	43	23	46	106
